# Biomaterials in personalized drug delivery: innovations, challenges, and future directions

**DOI:** 10.7717/peerj.20982

**Published:** 2026-03-19

**Authors:** Adna Hrapović, Nadia Islam, Asmaa Al Bourghli, Abas Sezer, Boris Kovalenko, Haris Lokvančić, Muhamed Adilović, Jasmin Šutković, Altijana Hromić-Jahjefendić, Vladimir N. Uversky

**Affiliations:** 1 International University of Sarajevo, Sarajevo, Bosnia and Herzegovina; 2 Department of Molecular Medicine, University of South Florida, Tampa, FL, United States of America

**Keywords:** Biomaterials, Drug delivery, Nanotechnology, Biocompatibility, Regenerative medicine, Healthcare applications, Personalized drug delivery, Nanomaterials

## Abstract

The growing global demand for effective and safe therapeutics has accelerated advances in biomaterials for drug delivery applications. Biomaterials, including polymers, metals, ceramics, and composites, play a central role in modern medical devices and therapeutic systems by enabling controlled interactions with biological environments. Initially defined as inert materials interfacing with biological systems, biomaterials are now rationally engineered to treat, replace, or evaluate tissue and organ functions. Recent progress in regenerative medicine, nanotechnology, and precision healthcare has expanded their use in drug delivery, where tunable physicochemical properties—such as degradation kinetics, surface chemistry, and mechanical stability—allow controlled release, protection of labile therapeutics, and enhanced accumulation at target sites. Polymer-based biomaterials enable sustained drug release through diffusion-controlled, degradation-mediated, or stimulus-responsive mechanisms, thereby extending therapeutic exposure and reducing systemic dosing frequency compared with conventional formulations. Nanostructured carriers, including liposomes, micelles, and dendrimers, further enhance drug delivery by improving solubility, cellular uptake, and site-specific targeting *via* size control, surface functionalization, and ligand-mediated interactions. Despite these advances, clinical translation remains limited by challenges related to immune–biomaterial interactions, batch-to-batch variability, long-term biodegradation behavior, and the scalability of manufacturing under regulatory constraints. Future biomaterial development must therefore emphasize precision fabrication, good manufacturing practice–compatible production, and biologically informed design strategies that account for patient-specific variability. This review provides a focused overview of biomaterial-based drug delivery systems, summarizes recent technological advances, and critically discusses mechanistic and translational challenges, including immune compatibility, degradation control, and regulatory compliance, with particular emphasis on their implications for personalized drug delivery.

## Introduction

In the last 20 years, biomaterials—including polymers, metals, ceramics—and composites have been developed and proposed as biological carriers. Different types of biomaterials play a crucial role in developing medical devices that enhance the quality of life. A huge number of literature reviews and research articles have addressed the biomaterials shortcomings, discussed their basic classifications, and biomedical applications. In general, biomaterials are traditionally defined as any materials that can be used in medical devices for interacting with biological systems (Chandel, Parihar & Khan, 2025). This definition has evolved to encompass materials designed to treat, evaluate, or replace tissue or bodily functions. Traditional drug delivery methods carry common limitations such as poor bioavailability, systemic toxicity, and lack of targeted release (Adepu & Ramakrishna, 2021). Due to their biodegradability, non-toxicity, and biocompatibility, biomaterials are highly effective in managing and delivering drugs directly to target tissues or organs (Oleksy, Dynarowicz & Aebisher, 2023; Wang, 2023). However, significant challenges remain, in particular to increase the targeting, decrease toxicity, and translate preclinical successes into clinical practice (Gao et al., 2023). A critical factor in biomaterials is their biocompatibility, which ensures that the materials do not harm patients, as per FDA standards (Zadpoor, 2020; Marin, Boschetto & Pezzotti, 2020). The development of regenerative medicine science, nanotechnology, and personalized healthcare systems has accelerated the development of biomaterial-based drug delivery systems. Most of these systems offer controlled drug release, site-specific delivery, and improved patient compliance. However, some questions about their scalability, regulatory approval, and long-term safety remain unclear. While earlier reviews have provided broad overviews of biomaterials and their applications (Chen et al., 2024; Harimoto, Jung & Mooney, 2025), and several comprehensive studies have critically examined biomaterial-based delivery systems in relation to therapeutic needs and clinical translation, most of these works focus on platform-level optimization rather than true patient-level personalization. Limited attention has been paid to how interpatient biological heterogeneity—such as differences in immune status, disease microenvironment, and molecular profiles—can be systematically integrated into the design, manufacturing, and deployment of biomaterial-based drug delivery systems. Therefore, despite the apparent saturation of the field, a critical personalization gap remains. This review addresses this gap by focusing on biomaterials through the lens of true personalization, highlighting how patient-specific biological parameters, immune stratification, and precision manufacturing strategies can be incorporated into drug delivery design. By synthesizing recent technological advances and case studies, this work aims to move beyond generalized solutions and contribute a more integrated framework for personalized biomaterial-based drug delivery, while also discussing persistent challenges related to biocompatibility, biodegradation, scalability, and regulatory pathways (El-Tanani et al., 2025; Zheng et al., 2025b).

## Survey Methodology

This review followed a structured and transparent literature search strategy designed to ensure reproducibility and minimize selection bias. Four electronic databases—PubMed, Scopus, Web of Science, and Google Scholar—were systematically searched between January and March 2025. The search strategy combined controlled vocabulary and free-text keywords related to biomaterials and drug delivery, including: (“biomaterials” OR “nanomaterials” OR “polymeric carriers”) AND (“drug delivery” OR “controlled release” OR “targeted delivery”) AND (“biocompatibility” OR “biodegradability” OR “immune response” OR “personalized medicine”). The initial search yielded 1,284 records across all databases. After removal of 312 duplicate records, 972 unique articles remained for title and abstract screening. At this stage, articles were excluded if they were unrelated to drug delivery applications, focused solely on non-biomedical materials, or lacking experimental, clinical, or mechanistic relevance. 238 articles were selected for full-text evaluation. Of these, 89 studies were excluded due to insufficient methodological detail, lack of relevance to biomaterial-based delivery systems, or focus on non-personalized therapeutic applications. An additional 27 articles were excluded because they were conference abstracts, opinion pieces, or non-peer-reviewed sources. The final dataset consisted of 149 peer-reviewed articles, including experimental studies, clinical investigations, and high-quality review papers published in English between 2012 and 2025. Reference lists of relevant review articles were manually screened to identify additional eligible studies not captured in the database search. To mitigate publication bias, studies reporting limited efficacy, adverse effects, immunogenicity, or translational barriers were intentionally retained. While publication bias cannot be fully eliminated, the use of multiple databases, explicit inclusion criteria, and manual reference screening strengthens the robustness and transparency of the review.

## Biomaterials and Their Role in Medicine

Advancements in regenerative medicine and tissue engineering have led to a significant rise in the utilization of biological materials for medical applications in the past decade. These materials, known as biomaterials, may originate from living organisms or be synthetically engineered (Wang, 2023). They are classified as natural (*e.g.*, cellulose, gelatine, alginate, chitosan, fibronectin) or synthetic (*e.g.*, poly(lactic-co-glycolic acid) (PLGA), polylactic acid (PLA), polycaprolactone (PCL)) based on their origin (Eldeeb, Salah & Elkasabgy, 2022; Rijal, 2023). Biomaterials offer various advantages such as biocompatibility, biodegradability, and non-toxicity, making them highly effective in enhancing biological functions, supporting damaged tissue, and ultimately improving the quality of life (Williams, 2014; Todros, Todesco & Bagno, 2021). They are used in a wide range of applications, including tissue engineering implants, human tissue healing, drug delivery systems, and biosensors (Cicha et al., 2022).

Despite their potential, the complexity of biomaterials poses a significant challenge to researchers and clinicians aiming to enhance the quality of life of patients with diverse medical conditions (Azevedo & Mata, 2022). Challenges such as potential toxicity, aggregation tendencies, shelf life, and other factors influencing the biomaterial’s effectiveness and safety limit the efficacy of current drug delivery systems (Drabczyk et al., 2024). Developing drug carriers with properties like non-toxicity, biodegradability, and adjustable size and shape will enhance targeted drug delivery while minimizing side effects (Adeosun et al., 2020). Interdisciplinary collaboration across fields like chemistry, biology, medicine, physics, engineering, materials science, and biotechnology is necessary for the development of these materials. Ensuring biomaterials meet biological requirements to prevent immune responses, as well as possessing suitable mechanical properties and corrosion resistance, are essential considerations (Eliaz, 2019).

### Clinical translation

The successful clinical translation of these drug delivery systems depends purely on demonstrating scalability, reproducibility, safety, and regulatory compliance (El-Tanani et al., 2025). In fact, the slow translation of drug delivery systems using nanoparticles is due to the combination of safety issues, biological complexity, manufacturing processes, legal obstacles, and high cost of production. Although nanomaterials hold great promise, approving them for the market required overcoming these challenges. For example, nanomaterials often have unique surface properties, shapes, and sizes that may interact unpredictably with biological systems (Albanese, Tang & Chan, 2012). Some of the mentioned issues are solved with surface modifications. Further, long-term toxicity, accumulation in organs (*e.g.*, liver, spleen), and unclear biodegradation pathways raise concerns in the scientific world (Jakic et al., 2024). In addition, regulators (FDA, EMA) require extensive safety data that is harder to generate than for conventional drugs, slowing down their approval for clinical use (Csóka et al., 2021).

### Surface modifications of biomaterials

Surface modification of biomaterials is a vital procedure to address inadequate surface properties, such as adhesion, adsorption, and biocompatibility prior to application (Zhou et al., 2019). The body’s immune response may lead to rejection of implanted biomaterials, while infections from microbial contamination can contribute to biomaterial dislocation and failure (Moshaverinia et al., 2013). Overcoming these challenges is essential, underscoring the importance of surface modification strategies (Moshaverinia et al., 2013; Zhou et al., 2019). Despite the rapid growth in biomaterial application and demand, challenges persist in production and safety, with biocompatibility and mechanical properties being crucial considerations (Bidarra, Barrias & Granja, 2014). Failures related to biocompatibility present significant challenges, influenced by factors like shape, size, intended use, and duration of application. Corrosion and ionization of implants due to tissue reactions and the corrosive physiological environment are commonly observed in biomaterial failures (Xing et al., 2016).

### Bioactive coating as a solution in drug delivery of nanomaterials

Bioactive coatings play a critical role in medical device design by enhancing the bonding between implants and living tissues. These coatings facilitate stem cell differentiation into osteoblasts, bone ingrowth, and enhance implant integration (Ercan et al., 2018). The first bioactive coating came in the form of a heparin-modified surface polymer blood oxygenator part, approved by the FDA in 1997 (Zhang, 2016). Furthermore, the recent development of biodegradable polymers as bioactive coatings has aimed to promote tissue formation, wound healing, and prevent infections (Raucci et al., 2020). A group of researchers showed that the biodegradable coatings based on PLGA are efficiently used in surface modification of medical devices, such as metallic implants and wound dressings (Gherasim et al., 2021). Recently, in 2024, a study demonstrated a significant effect of a metal oxide coating, enhancing the properties of Co-Cr-Mo dental alloy (Łosiewicz et al., 2024).

A variety of techniques are used for bioactive coating on metal implants, each differing in how they deposit material and in the resulting surface properties. Solution-based processes, including dip coating, drop casting, electrospinning, sol–gel + electrodeposition, and electrophoretic decomposition, allow uniform coverage at low temperatures and are suitable for incorporating bioactive molecules (Kravanja & Finšgar, 2022). Vapor-based techniques, including chemical vapor decomposition and physical vapor decomposition methods, generate dense, highly adherent films with precise thickness control (Kravanja & Finšgar, 2022; Saba, Saad & Rashid, 2024). Plasma spraying and biomimetic decomposition produce thicker, often porous coating that enhances osseointegration (Daugaard et al., 2010; Kravanja & Finšgar, 2022). 3D printing enables precise control of implant micro-architecture, allowing for improved biological performance (Yücer, Sarac & Ciftci, 2025).

## Biomaterials for Drug Delivery

Drug delivery is a critical aspect of the pharmaceutical industry that aims to precisely administer therapeutic agents to specific target sites in the body (Adeosun et al., 2020). The concept of a “magic bullet” introduced by Paul Ehrlich (Schwartz, 2004) highlights the ideal drug delivery system that selectively targets diseased cells without affecting healthy ones.

Therefore, biomedical engineers have made significant contributions to understanding drug delivery barriers and advancing novel drug delivery technologies. These technologies encompass various routes of drug administration and delivery vehicles, such as nanoparticles and lipid nanoparticles, to ensure effective drug delivery and protection of therapeutic agents from degradation (Tewabe et al., 2021; Xu et al., 2022; Liu et al., 2023). Engineering advancements like microneedle patches for painless vaccinations and innovative nanoparticle designs show promise for improving drug delivery methods and enhancing treatment outcomes (Menon et al., 2021). Additionally, optimized drug delivery vehicles, like nanoparticles loaded with anti-inflammatory agents for ailments such as acute respiratory distress syndrome (ARDS), hold the potential for targeted and effective therapies (Menon et al., 2021; Prasanna et al., 2021; Dhege, Kumar & Choonara, 2024).

Nanoparticles revolutionize drug formulation and delivery by leveraging nanotechnology, a multidisciplinary field that manipulates materials at the molecular level. With sizes ranging from 100 to 500 nm, nanoparticles can be tailored to deliver drugs to specific tissues, minimizing toxicity and enhancing treatment efficacy, notably in cancer therapy (Park et al., 2016). Through injection, inhalation, or oral intake, nanoparticles interact with proteins in the body, facilitating drug distribution to organs *via* blood capillary absorption and lymphatic elimination (Arakha et al., 2021). To evade immune activation and improve targeting, nanoparticle size and surface characteristics are crucial, with an optimal size of around 100 nm for efficient drug delivery and BBB traversal. Small nanoparticles exhibit faster drug release and reduced immune response, underscoring their potential to improve therapeutic outcomes (Sykes et al., 2016).

Effective drug release from nanoparticles hinges on various factors like pH, drug solubility, temperature, and diffusion processes within the nanoparticle matrix (Modi & Anderson, 2013). To enhance biocompatibility and circulation time, hydrophilic surfaces, often coated with polymers like polyethylene glycol (PEG) or polyoxamer, are employed to prevent protein binding and premature drug loss. Erosion of the nanoparticle matrix facilitates controlled drug release, with the addition of auxiliary agents such as poly(ethylene oxide) and poly(propylene oxide) (PEO-PPO) mitigating early drug release due to polymer-drug interactions (Lee & Yeo, 2015). Nanoparticles offer targeted drug delivery capabilities to damaged tissues through specific ligand coatings like peptides, antibodies, or proteins, minimizing off-target effects on healthy tissues (Rizvi & Saleh, 2018). For instance, nanoparticle-based delivery systems can precisely deliver chemotherapeutic agents to tumor sites, reducing systemic toxicity and preserving normal tissues. Micelles and liposomes are other vehicles for localized chemotherapy delivery (Shen et al., 2016).

## Nanotechnology in Biomaterials

Nanotechnology, a rapidly growing field for the past 50 years, spans various disciplines like physics, biology, pharmacy, electronics, chemistry, and medicine, focusing on materials within the range of (1–100) nm (Gowda et al., 2022). These nanomaterials possess unique properties that mimic extracellular matrix components, enabling direct delivery of active substances. Their high surface area to volume ratio grants them distinct characteristics, including enhanced mechanical properties and potential antiviral, antibacterial, and antifungal properties (Baig, Kammakakam & Falath, 2021). Nanoparticles can penetrate cell membranes, aiding in protein absorption and making them valuable for drug delivery and tissue regeneration applications (Zheng et al., 2021). Moreover, nanomaterials exhibit exceptional thermal and electrical conductivity, with the ability to transform non-magnetic materials into magnetic entities at the nanoscale level. Additionally, they offer biological advantages such as biocompatibility, low immunogenicity, and biodegradability (Gul et al., 2019; Vinod & Philip, 2022).

### Applications of nanomaterials in drug delivery

**Figure 1:**
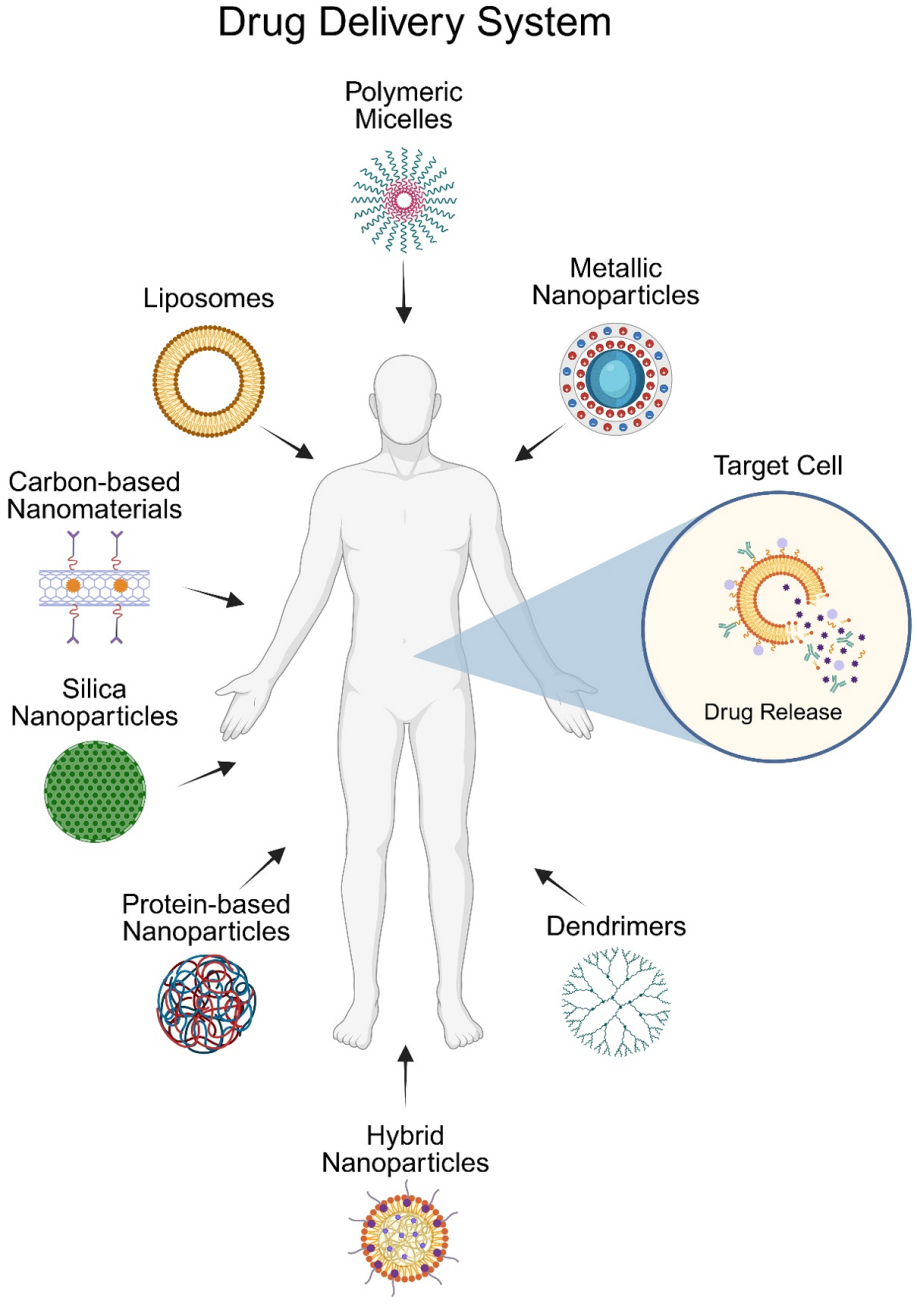
Graphical representation of application of nanomaterials.

Nanotechnology in drug delivery enhances therapeutic efficacy and minimizes side effects by utilizing nanomaterials as carriers for targeted delivery, reducing systemic distribution (Triantafyllopoulos & Papaioannou, 2022). These advancements are particularly crucial in cancer treatment, where precise drug delivery is vital. The summary of applications can be seen in Fig. 1 (Sezer, 2025).

Common nanomaterial drug carriers include liposomes, polymeric micelles, metallic nanoparticles, carbon-based nanomaterials, silica nanoparticles (mesoporous silica), protein-based nanoparticles, hybrid nanoparticles, and dendrimers (Acharya & Sahoo, 2011; Prajapati et al., 2019).

#### Liposomes

Liposomes, spherical vesicles with a phospholipid bilayer enclosing an aqueous space, fall within the size range of 0.01–5.0 µm, positioning them as nano drug delivery systems. They were the first nanoparticle type to receive approval for medical applications, offering unique properties like the ability to encapsulate both hydrophilic and hydrophobic molecules, enhancing drug delivery versatility. Comprising biocompatible and biodegradable materials, liposomes safeguard drugs from degradation, improve bioavailability, and allow for efficient drug encapsulation (Farooque, Wasi & Mughees, 2021). Moreover, their surface can be tailored to incorporate targeting ligands, antibodies, or functional groups for specific drug delivery purposes, making them ideal for targeted drug delivery, enhancing drug efficacy, and minimizing side effects (Forssen & Willis, 1998; Riaz et al., 2018). Further, liposomes in combination with local hyperthermia represent a promising tool for tumor-specific drug delivery (Chen et al., 2014). In this regard, understanding of the molecular basis of lipid functions needs a biophysical approach. A key feature of lipids is the liquid crystalline state of matter, represented in different phases and connected by phase transitions. The transition phase changes are influenced by temperature, ions, electric fields, and pH, causing significant changes in the physical properties and organization, affecting the functions of liposomes (Kinnunen, Alakoskela & Laggner, 2003). For example, with the modification of melting (transition) temperature, the liposomes containing various ratios of 1,2-dipalmitoyl-sn-glycero-3-phosphacholine (DPPC) and hydrogenated soy phosphatidylcholine (HSPC) are promising in the application in the field of thermosensitive liposomes (TSLs) (Chen et al., 2013).

A specific event is observed with liposomes, particularly in PEGylated liposomes (∼100 nm, without drug), when injected into the same animal twice, separated by some time, the second dose is cleared from the blood much faster than expected. This phenomenon, loss of long-circulating behaviour, is known as accelerated blood clearance (ABC) phenomenon (Ishida & Kiwada, 2008). Consequently, this leads to a diminished circulation time typically associated with PEGylation, thereby impairing the delivery efficiency and effectiveness of the nano-formulations (Pan et al., 2025). Moreover, the ABC phenomenon was also triggered by the subsequent intravenous administration of PEGylated solid lipid nanoparticles (PSLN) followed by an initial subcutaneous injection of PSLN, where the degree of accelerated clearance resulting from the subcutaneous injection was comparable to or even less than that observed with the intravenous injection first (Zhao et al., 2012).

Sui et al. (2023) compared linear and branched PEG lipid derivatives on cationic liposomes, used for photothermal therapy. Linear PEGs induced strong anti-PEG antibody responses (mainly IgM from B cells), and a clear ABC effect on subsequent doses. However, branched and cleavable branched PEG chains elicited much lower anti-PEG responses, did not significantly activate complement, and largely avoided ABC, while maintaining or improving therapeutic efficacy (Sui et al., 2023). In one recent study published in 2025, Zheng et al. (2025b), developed a “drug release reporter” PEGylated liposome containing an SN 38–GSH probe to quantify plasma-induced accelerated drug release (ADR) as an alternative for ABC. Zheng et al. (2025a) demonstrated that slow intravenous infusion of PEGylated liposomes (*vs* bolus) markedly reduced ABC in rats, likely by lowering instantaneous exposure and immune activation during the priming dose.

#### Polymeric micelles

Polymeric micelles, nanoscale structures resulting from the self-assembly of amphiphilic block copolymers in water, exhibit a core–shell architecture with dimensions ranging from 10 to 100 nm (Kuperkar et al., 2022). The hydrophobic core enables the encapsulation of poorly soluble drugs, while the hydrophilic shell imparts stability and biocompatibility. These systems have garnered significant attention in targeted drug delivery owing to their efficacy in enhancing drug retention within tissues, protecting drugs from enzymatic degradation, and facilitating cellular uptake (Ghosh & Biswas, 2021). Polymeric micelles can be formed only when the concentration of amphiphilic polymers is above the critical micelle concentration (CMC), the point at which self-assembly into micelles becomes thermodynamically favourable. If the concentration is below the CMC, dispersed polymer chains (unimers) are commonly found. On the other hand, if the concentration of micelles is high, the additional polymer tends to form micelles instead of increasing the concentration of unimers (Perumal, Atchudan & Lee, 2022).

During the drug delivery applications, the CMC serves as a practical measure of thermodynamic stability during dilution, and when diluting below the CMC, we observe a disassembly of micelles in the bloodstream. This is found *in vivo*, as injected formulations undergo substantial dilution in the bloodstream, therefore the lower CMC values typically indicate better micelle stability under physiological conditions (Hussein & Youssry, 2018). In comparison to small non-polymorphic surfactants, polymeric micelles generally show lower CMCs, resulting in greater resistance to dissociation caused by the dilutions (Kulthe et al., 2012). Further, the CMC is affected by the sole architecture of the polymers and the surrounding environment, as these elements change the free-energy behaviour between unimers and aggregates (Owen, Chan & Shoichet, 2012). In experimental environment, the CMC is often analysed by fluorescence probe techniques (*e.g.*: pyrene intensity ratios), and with other methods like surface tension measurements, with the threshold indicating micellization (Aguiar et al., 2003). Moreover, polymeric micelles possess biocompatibility and facile elimination pathways, thus minimizing toxicological risks. Particularly advantageous for highly toxic drug delivery, they are easily scalable and cost-effective to produce. In addition to oncology, polymeric micelles find application in various therapeutic domains, including infectious diseases, cardiovascular disorders, inflammation, and neurological conditions (Vinchurkar & Kuchekar, 2021).

#### Metallic nanoparticles

##### Iron nanoparticles.

Iron-based nanoparticles, particularly in the form of iron oxide nanoparticles (IONPs), have many great characteristics, such as large surface area and physicochemical characteristics, but what sets them apart from other nanoparticles are their magnetic traits, which enable precise control for targeted drug delivery (Batool et al., 2021; Graham et al., 2025). Leveraging a magnet enables the localization of these particles to specific treatment sites, thereby reducing the necessary dosage and minimizing systemic drug dispersion (Hirschbiegel et al., 2023). In addition, when exposed to an alternating magnetic field, IONPs can produce heat and kill cancer cells, as those cells are sensitive to heat. Furthermore, IONPs can produce reactive oxygen species (ROS), which can damage cancer cells (Vangijzegem et al., 2023). IONPs are usually coated with a hydrophilic substance, usually polyethylene glycol, to sustain the stability in aqueous solutions and to reduce the immunogenicity of these particles (Wang & Bai, 2023). One study showed that liposomes, biocompatible lipid-based nanoparticles, were co-loaded with IONPs and doxorubicin, a chemotherapy drug. These nanoparticles were applied on melanoma cells and have generated heat with laser induction. The inhibitory effect of these nanoparticles was superior to doxorubicin and photothermal therapy (PTT) (Park et al., 2023). Even with all the great potentials of IONPs, there are some limitations that restrict the use of iron nanoparticles (FeNPs), such as the difficulty of maintaining the nanoparticles in the desired organ after the removal of the magnetic field, in addition to the challenge of dealing with the three-dimensional environment of the body (Schneider-Futschik & Reyes-Ortega, 2021).

##### Gold nanoparticles.

Among metallic nanoparticles, gold nanoparticles (AuNPs) are notable as they can be synthesized with different sizes and shapes, including rods, cubes, spheres, and triangles, making each suitable for a different application (Eker et al., 2024). AuNPs can be used for several medical purposes, such as drug delivery, anticancer and antimicrobial agents, and tissue engineering (Georgeous et al., 2024). In addition, Gold nanoparticles have the ability to absorb light and rapidly convert it into heat, making them a great candidate for photothermal therapy (PTT). AuNPs target cancer cells in the body by different strategies, such as attaching ligands to their surfaces. Near infrared (NIR) light is directed at the tumor area and AuNPs convert it into heat, killing cancer cells with hyperthermia, without affecting normal cells (Badir, Refki & Sekkat, 2025; Shukla et al., 2025). Despite the diverse applications of AuNPs, they can cause cytotoxicity, particularly in the liver, spleen, and kidneys (Sani, Cao & Cui, 2021).

##### Silver nanoparticles.

Silver nanoparticles (AgNPs) have gained a lot of interest due to their unique chemical, physical, and biological properties, making them ideal for a variety of applications, including drug delivery, therapeutics, and diagnostics (Lee & Jun, 2019). They can be used as anti-diabetic and anti-cancer treatments, as well as vaccine adjuncts (Meher et al., 2024). Moreover, AgNPs are particularly important as they possess antimicrobial properties that are superior in comparison with their bulk forms and with other metallic nanoparticles (Bruna et al., 2021). However, it can be toxic. *In vitro* studies showed that AgNPs can be genotoxic because the uptake of AgNPs in cells produces reactive oxygen species and leads to oxidative stress. Furthermore, because of the small size of AgNPs, they can easily enter the living organism through skin, inhalation, and ingestion, causing damage to different vital organs such as the liver, the heart, and the respiratory system (Noga et al., 2023).

##### Polymeric nanoparticles.

Polymeric nanoparticles (PNPs) are widely used in drug delivery as they consist of biodegradable and biocompatible polymers (Prajapati et al., 2019). Because of their properties, they can encapsulate both hydrophilic and hydrophobic drugs and protect them from degradation. They can also be loaded with one drug or a combination of drugs (Sánchez, Mejía & Orozco, 2020). Furthermore, they can be used for targeted delivery and controlled release, and reduce side effects (Eltaib, 2025). Nevertheless, there are several limitations to PNPs related to toxicity, stability, and production, which require further study to understand the cytotoxicity mechanism and improve the manufacturing process (Eltaib, 2025). Among PNPs, chitosan nanoparticles (CNPs), Poly(lactide-co-glycolide) (PLGA) nanoparticles, and polyethylene glycol (PEG) are some of the most distinct. Chitosan is a polysaccharide that is biodegradable, biocompatible, and has low toxicity. CNPs have a diverse range of applications, including drug delivery and cancer therapy (El-Naggar et al., 2022). CNPs are quite promising in cancer therapy as they reduce cytotoxicity to normal cells and overcome drug resistance in different cancer models (Garg et al., 2019). PLGA is another biodegradable and biodegradable polymer used as a nanocarrier enabling targeted therapy and controlled release. They can be used in a variety of conditions, including cancer treatment, gene delivery, tissue regeneration, and in neurodegenerative disease management (Omidian, Wilson & Castejon, 2025). PNPs can be specifically coated by some stabilizers, such as PEG, in this example for the zeta potential applications (Zielińska et al., 2020). This surface coating eventually can help evade the immune system, prolonging the time the nanoparticles remain in the body and enhancing the delivery of drugs to targets (Suk et al., 2016; Mukherjee et al., 2019).

Polymeric nanoparticles often show two extreme behaviours, bulk erosion and surface erosion. Those characteristic behaviour patterns are primarily influenced by the interplay between water penetration (diffusion) and the cleavage of polymer bonds (degradation rate) (Burkersroda, Schedl & Göpferich, 2002). Bulk-eroding nanoparticles, commonly found in PLGA systems, where the water absorption throughout the particle is faster than the polymer chains can be cleaved, causing a degradation that occurs throughout the entire volume. This can cause the formation of porosity and start autocatalytic effects within the interior (Makadia & Siegel, 2011).

On the other hand, surface-eroding nanoparticles mainly degrade at their outer layers, because the surface reaction occurs at a quicker rate than water can move into the polymer. Accordingly, the nanoparticles hold a dense core while their radius gradually decreases over time (Stiepel et al., 2022). This difference is important because bulk erosion typically results in more complex, time-dependent release patterns, while surface erosion can result in a more predictable, surface-controlled mass loss under favourable conditions (Makadia & Siegel, 2011).

#### Dendrimers

Dendrimers are polymers that consist of tendrils surrounding a hollow core. They can be used as drug carriers because of their properties. The drug is loaded into particles, and since they either contain polyethylene glycol (PEG) or have a PEGylated surface, this modification reduces immunogenicity. Therefore, the drug’s residence time in the body is extended, and its toxicity is minimized (Patel et al., 2020). Among the main types of dendrimers, polyamidoamine (PAMAM), polypropyleneimine (PPI), polyamide-, polyether-, polyester-, and phosphorous-based dendrimers are distinguished (Wang et al., 2022). Chauhan (2018) describes the main advantages of dendrimer-based drug delivery systems as well as their ability to increase water solubility, stability, dissolution, drug release, targeting, and pharmacokinetics of various drugs. An interesting experimental study by Yang et al. (2019) shows how, with the assistance of co-modification of PEGylated PAMAM dendrimers with cyclic RGD hexapeptide and penetratin, the goal of noninvasive targeting and penetration of the ocular posterior segment can potentially be achieved (Yang et al., 2019). It should be noted that despite the above-mentioned advantages of dendrimers, their use is still limited due to the disadvantages, such as high production costs and toxicity (Surekha et al., 2021).

#### Carbon-based nanomaterials

Among the carbon-based nanomaterials, carbon nanotubes, graphene oxide, and fullerenes should be described. When discussing carbon nanotubes, it should be noted that they can be functionalized to ensure biocompatibility and conjugated with drugs or contrast agents. In the first case, they are promising drug delivery systems, and in the second, they can potentially assist in visualization during diagnostic procedures. The use of carbon nanotubes can significantly improve the mechanical and electrical properties of hydrogels as drug delivery systems, but it comes with a number of potential risks. For example, in some cases, part of the carbon nanotube (CNT) may remain and accumulate in the body, and the consequences of such accumulation are unknown (Simon, Flahaut & Golzio, 2019; Komane et al., 2020). Two-dimensional reduced graphene oxide is also a very promising biomaterial. It has potential applications as an agent in photothermal cancer therapy due to its photothermal conversion in the IR range, as well as a drug delivery vehicle because it can be functionalized with, for example, photosensitizer molecules. The main advantages of reduced graphene oxide as a drug delivery system are its high biocompatibility and targeted delivery (Dash et al., 2021). Despite these promising advantages, graphene oxide should be considered as a therapeutic option with caution, as its use is associated with a number of potential risks, such as pulmonary toxicity, oxidative stress, or thrombotoxicity (Qu et al., 2018). Fullerene C60, which is one of the allotropic modifications of carbon, is interesting in terms of its ability to form clathrates with small molecules. Thus, it can potentially be used to remove toxins (such as herbicides that have entered the body with food) during the treatment of poisoning (Nozdrenko et al., 2021). Information on the toxicity of fullerenes contained in the literature is contradictory, but it must be taken into account when considering them as therapeutic agents and delivery systems (Bolshakova, Zherebyatieva & Sarantseva, 2025).

#### Silica nanoparticles (mesoporous silica)

Mesoporous silica nanoparticles (MSNs) are promising delivery vehicles for drugs and contrast agents because they combine the chemical and physical stability of silica with the potential offered by the network of cavities in a mesoporous structure. MSNs can be synthesized with controlled size, they can be easily functionalized, and are biocompatible (Manzano & Vallet-Regí, 2020). MSNs are particularly promising for the delivery of poorly water-soluble drugs, as they increase their apparent solubility (Maleki et al., 2017). By ensuring controlled drug release, silica nanoparticles can enhance the therapeutic activity of drugs. For example, Kundu and co-authors describe an increase in the antitumor potential of umbelliferone using this method (Kundu et al., 2020). It should be noted that the toxicological profile of silica nanoparticles includes a number of possible harmful effects on living organisms, such as cellular stress, necrosis, and genotoxicity (Liu & Sayes, 2022).

#### Protein-based nanoparticles

Protein nanoparticles based on, for example, fibroin, albumin, gelatin, gliadin, or bobumin are potentially a good alternative to synthetic drug delivery systems. Obviously, their main advantages are biocompatibility and biodegradability (Hong et al., 2020). A classic example of an FDA-approved drug that uses protein-based nanoparticles as a delivery system is Abraxane^®^ (Celgene), which is a paclitaxel nanoparticle bound to albumin that is used to treat breast cancer. This drug has improved solubility and targeted delivery to the tumor (Choi & Han, 2018). Despite their obvious advantages, protein-based nanoparticles are not without drawbacks. For example, proteins, as natural polymers, are difficult to produce with controlled and reproducible characteristics (primarily molecular weight), which complicates their mass production (Kianfar, 2021). It should also be noted that foreign proteins introduced into the human body can potentially cause an immune response (Wang et al., 2020a).

#### Injectable biomaterials

Evaluation of injectable biomaterials in regenerative medicine offers promising advancements, providing less invasive alternatives to conventional treatments (Wang, 2023). These biomaterials can be derived from natural sources, synthetic materials, or their composite combinations (Eldeeb, Salah & Elkasabgy, 2022; Wang, 2023). This approach is particularly beneficial for treating vertebral fractures, tumor resections, and craniofacial defects, facilitating accelerated repair processes (Oleksy, Dynarowicz & Aebisher, 2023). Injectable biomaterials are versatile for various clinical applications, enabling targeted delivery of bioactive molecules and growth factors to specific tissues (Eldeeb, Salah & Elkasabgy, 2022). Meeting diverse treatment needs has led to the development of formulations and functionalities of injectable biomaterials.

Injectable biomaterials offer several advantages, such as invasive delivery at minimal points, reducing patient discomfort and recovery time (Gu et al., 2025); enhanced tissue integration, as they seamlessly integrate with host tissues and promote natural healing processes (Chen et al., 2025); controlled drug release, since injectable systems can be engineered for controlled and sustained release of therapeutic agents, thereby improving treatment efficacy (Shen et al., 2025); and personalization and optimization, as these biomaterials can be modified with bioactive molecules to enhance specific cellular responses (Martinet et al., 2025).

Commonly used materials in medical research for injectable biomaterials include alginate, collagen, gelatine, chitosan, fibrin in hydrogel or microsphere forms, as well as bioactive glasses, calcium phosphates, and polymethyl methacrylate (PMMA) in cement or paste formulations (Bharadwaj, 2021; Zhang et al., 2022; Socci et al., 2023; Xie et al., 2025). For example, a recent study demonstrated that fibrin in a hydrogel can infiltrate submucosal tissue, effectively reducing postoperative swelling and pain, while also promoting faster wound healing (Aliberti et al., 2025). Another study, using osteoporotic porcine vertebrae, demonstrated that the low-modulus PDMS-containing PMMA bone cement significantly delayed adjacent fractures compared to conventional PMMA bone cement, which turns out to be a safer and more effective treatment option for osteoporotic vertebral compression fractures (Kim et al., 2025).

Hydrogels, a key biomaterial category, possess the ability to adapt their properties in response to biological recognition events, such as nutrient presence or enzyme activity. Comprised of up to 90–99% water, hydrogels form elastic networks through cross-linked polymers, offering biocompatibility and controllable swelling behavior (Ulijn et al., 2007). These versatile materials can be designed using natural or synthetic components and have found diverse applications in medicine, including as adhesive cardiac patches for improved heart function restoration (Iravani & Varma, 2022).

Hydrogels are commonly used in soft contact lenses to allow gas diffusion while maintaining moisture on the eye’s surface. In medical applications, hydrogel patches support tissue regeneration by creating a protective barrier for damaged tissues, promoting wound healing, and serving as depots for bioactive agent delivery (Mandal et al., 2020). Microparticles, another significant drug delivery system, offer precise drug release, protection, and easy administration (Siepmann & Siepmann, 2006). These microparticles can be customized in size and morphology for various therapeutic needs, making them ideal for pulmonary drug delivery and the administration of insulin, corticosteroids, and chemotherapy agents (El-Sherbiny, El-Baz & Yacoub, 2015). The interdisciplinary study of biomaterials by chemists, physicists, biologists, and pharmaceutical scientists is crucial for the development of innovative treatment and diagnostic techniques. The future of biomaterials in drug delivery holds promise for personalized and safer therapies through the manipulation of molecular sizes and surface properties to enhance drug delivery efficiency (Rizvi & Saleh, 2018).

#### Hybrid nanoparticles

Recently, an approach involving the synthesis of hybrid nanoparticles that combine the advantages of several of the above-mentioned (and other) drug delivery systems has become popular. A classic example of hybrid nanoparticles is lipid-polymer particles, consisting of a polymer core and a shell representing a lipid layer. They combine the properties of polymer carriers, such as stability and the possibility of artificial synthesis, with those of liposomes, such as bioavailability and safety (Mukherjee et al., 2019). Other examples of hybrid nanoparticles are composites of carbon nanotubes with biomolecules. The advantages of CNTs have been discussed above, but their practical application in medicine is limited by their hydrophobicity and poor biocompatibility. This problem can be solved by obtaining their conjugates, for example, with proteins or lipids (Anaya-Plaza et al., 2021). Hybrid nanoparticles are synthesized in order to overcome the shortcomings of simpler types of carriers through their multifunctionality. In turn, their main disadvantage is the relatively high complexity of their structure, which can potentially cause difficulties in their production.

In Table 1, we present several examples of commonly used injectable biomaterials and their applications in regenerative medicine.

**Table 1:** Summary of main sections of biomaterials for drug delivery.

**Injectable biomaterial**	**Role and function**	**FDA status**	**Advantages**	**Disadvantages**	**References**
Alginate	Natural polymer used as a hydrogel matrix; provides structural support and cell encapsulation; used in drug delivery and tissue regeneration	Approved in wound dressing and cell encapsulation	Mild gelation, high biocompatibility	Poor cell adhesion, limited mechanical strength	Tan et al. (2010); Lee & Mooney (2012); Bidarra, Barrias & Granja (2014); Bharadwaj (2021)
Collagen	Major extracellular matrix protein; promotes cell adhesion and wound healing	Approved in wound healing and dermal fillers	Excellent bioactivity, promotes cell adhesion and tissue integration	High cost, rapid degradation	Xing et al. (2016); Sen et al. (2025); Huang et al. (2025)
Gelatin	Denatured form of collagen; controlled drug release and scaffolding	Approved in certain derivatives (gelatin sponges)	Biodegradable, low toxicity	Weak mechanical properties	Lukin et al. (2022); Chalard et al. (2024)
Chitosan	Natural polysaccharide from chitin; supports tissue regeneration and hemostasis	Approved in hemostatic and wound applications	Antibacterial, biodegradable, promotes healing	Solubility issues	Garg et al. (2012); Kantak & Bharate (2022); El-Naggar et al. (2022); Li et al. (2024); Wang et al. (2024)
Fibrin (hydrogel/microspheres)	Blood-clotting protein used as biomaterial; promotes cell migration and vascularization in wound and bone healing	Approved as sealants and surgical adhesives	Strong bioactivity, supports angiogenesis	Rapid degradation, high cost	Lorentz et al. (2011); Ceccarelli & Putnam (2014); Roberts et al. (2020); Vach Agocsova et al. (2023); Aliberti et al. (2025)
Hydrogels (synthetic/natural)	Versatile polymer networks	Approved depending on polymer	Controlled drug release, minimally invasive		Dreiss (2020); Ranamalla et al. (2024); Segneanu et al. (2025); Feng et al. (2025)
Calcium Phosphates (*e.g.*, HA, TCP)	Inorganic bone-mimicking materials; bone regeneration	Approved in bone graft substitutes	Excellent bone integration	Limited injectability without additives	Eliaz & Metoki (2017); Razavi et al. (2020); Wei et al. (2020); Sabouri et al. (2026)
PMMA (Polymethyl methacrylate)	Synthetic polymer cement; bone fixation and dental applications	Approved for orthopedic and dental use	High mechanical strength	Non-degradable, exothermic polymerization	Hacker & Mikos (2011); Bistolfi et al. (2019); Kim et al. (2025)

## Biodegradable and Bioresorbable Biomaterials: Regulation, Ethics, Biocompatibility, and Immunomodulation

Biomaterials, essential in medical applications, must exhibit biocompatibility to avoid adverse host reactions. Compliance with FDA guidelines ensures patient safety (FDA). These materials must also fulfill diverse design criteria encompassing mechanical robustness, geometry, and electrical properties (Zadpoor, 2020). Historical progression categorizes biomaterials into inert (1960–1970), bioactive (1980–1990), biodegradable (2000–2010), and smart biomaterials (2010-present). Inert biomaterials aimed at tissue replacement, while bioactive counterparts enhanced device efficacy through coatings. Biodegradables addressed infection risks prevalent in bioactives by promoting absorption within the body, obviating the need for replacement surgeries. The current era of biomaterials focuses on mimicking natural tissue structures to facilitate tissue repair. Despite advancements, first-generation biomaterials remain prevalent in clinical practice (Zadpoor, 2020; Marin, Boschetto & Pezzotti, 2020; Todros, Todesco & Bagno, 2021).

Biodegradable and bioresorbable biomaterials enable gradual degradation within the body, facilitating tissue regeneration without necessitating replacement. Of note, magnesium alloy emerges as a promising candidate for bioresorbable applications, although concerns regarding its use in implants exist (Gv & Setti, 2021). As corrosion progresses, hydrogen gas evolution can accumulate at the implant site, creating microbubbles that further disrupt structural stability and surrounding tissues. Mechanical failure often follows through crack initiation and propagation along corrosion-affected zones, ultimately resulting in premature fracturing or collapse of the implant before therapeutic goals are achieved. These failure pathways underscore the need for surface modification, alloying strategies, and controlled degradation approaches to enhance the reliability of magnesium-based biomaterials in drug delivery applications (Noviana et al., 2016). Scaffold engineering employs a range of biodegradable materials such as fossil-based polymers, poly (*ɛ*-caprolactone), poly(vinyl) alcohol, polyethylene glycol, polypropylene fumarate, polyurethane, modified polyurethanes, collagen, hyaluronic acid, chitosan, and fibrin (Vach Agocsova et al., 2023). Biomaterials have revolutionized drug delivery systems, enhanced targeted therapy, and mitigated the adverse effects of conventional chemotherapy on healthy tissues. Notably, polyurethane drug delivery systems show promise in cancer treatment, offering a novel approach that instills optimism and novel prospects for cancer patients (Sobczak & Kędra, 2022).

Bone tissue engineering is a rapidly advancing field, emphasizing biodegradable biomaterials over non-degradable counterparts. Traditional biodegradable materials encompass metals, polymers, and ceramics, offering long-lasting treatments (Shekhawat et al., 2021). Recent innovations include intelligent micro-nano materials and cell-based products, minimizing the need for repeat surgeries and reducing healthcare costs. Biodegradable ceramics, derived from natural clay and other components, play a crucial role in tissue repair, bone defect filling, and fracture healing. Hydroxyapatite, tricalcium phosphate, and dicalcium phosphate are commonly used ceramics, valued for their corrosion resistance, biocompatibility, and biological activity, aiding in gradual tissue regeneration (Razavi et al., 2020; Wei et al., 2020).

Biomaterial development necessitates compliance with international and country-specific regulations and ethical guidelines. Following these standards ensures the efficacy, safety, and responsible utilization of biomaterials in various applications. Key guidelines include adherence to international standards and regulatory guidance, such as global risk classification and access to medical device standards. Compliance with ethical considerations in preclinical animal and clinical human research is paramount. Addressing ethical challenges related to embryonic and fetal-derived tissues and gene therapy is essential. Ensuring informed consent, privacy protection, equitable access, affordability, environmental sustainability, and cultural sensitivity are critical aspects in the ethical development and application of biomaterials. Collaboration among diverse stakeholders is imperative for the establishment of universally recognized ethical regulations in this field (Schuh & Funk, 2019; Razavi et al., 2020; Hyun, Scharf-Deering & Lunshof, 2020; Sekar et al., 2021; Hunckler & Levine, 2022).

In the context of immune response, maintaining immune homeostasis is crucial for defending against infections and managing tissue development, regeneration, and repair. A key focus in current research is on immunomodulation to enhance tissue regeneration and control immune responses. Immunomodulatory biologics, such as antibodies and drugs, have been developed to modulate immune activity in different conditions. However, challenges exist in their systemic administration, including short half-life, lack of targeting ability, and potential adverse reactions. Biocompatibility is a critical aspect of biomaterial development, ensuring that materials do not induce harmful effects in the body. Biocompatibility testing, required by regulatory bodies, is essential for various biomaterials used in medical applications, such as dental implants and prostheses. Enhancing resistance to bacterial infections is a key focus in biomaterial development to reduce hospital-acquired infections. Surface chemistry plays a significant role in biocompatibility, influencing cell adhesion, protein absorption, inflammatory response, antimicrobial properties, and biodegradation of biomaterials.

Since the 1970s, biodegradable polymers have been developed and have become widely accepted as an efficient system to deliver drugs (Zhu et al., 2022). For example, the synthetic biodegradable polymers, polyesters, are widely used due to their biodegradability, biocompatibility, and ease of processing (Jain et al., 1998). Among several polyesters, poly(lactide-co-glycolide) (PLGA) is the most popular, used in microparticle systems. PLGA degrades *via* hydrolysis of ester bonds, with the encapsulated drug released by bulk erosion mechanisms (Liu et al., 2020). Manipulating surface chemistry can regulate cell-material interactions and degradation rates, contributing to the overall success of biomaterials in medical applications (Ghasemi-Mobarakeh et al., 2019; Zhang, Mou & Jiang, 2020; Raut et al., 2020; Jurak et al., 2021; Bu et al., 2022; Chu et al., 2023; Bandyopadhyay et al., 2023; Zhang et al., 2023).

## Synthesis of Biomaterials and Their Basic Morphological Characteristics

The methods of synthesis of biomaterials directly influence their main morphological characteristics, which in turn determine the possible applications of each biomaterial. In Table 2, we summarize all common scaffolds and their examples in relation to biomaterials.

**Table 2:** List of scaffolds used for drug delivery systems and clinical purposes.

**Types of scaffolds**	**Materials**		**Example**	**Application**	**Reference**
Natural polymer scaffolding	Protein	Collagen	-Tropocollagen nanofibers	Bone regeneration, matrices for cell growth, skin replacements	Shih et al. (2006); Raftery et al. (2019)
		Silk	-Silk fibroin (SF) films and hydrogels, silk porous 3D scaffolds	Neuro-engineering, stroke, and traumatic brain injury-related disorders	Martín-Martín et al. (2019)
	Polysaccharides	Hyaluronic acid (HA)	-HA-based hydrogels	Tissue engineering, wound healing	Chung & Burdick (2009); Unterman et al. (2012)
		Alginate	-Alginate hydrogels	Drug delivery, wound healing	Kuo & Ma (2001); Wong (2004); Sun & Tan (2013)
	Natural composite scaffolds	Collagen-based	-Collagen-glycosaminoglycan (chondroitin-6-sulfate) (Coll-GAG) -Collagen hydrogel-scaffold -Collagen-Hyaluronic Acid (CHyA) -Collagen-Hydroxyapatite (CHA) -Ceramic-collagen composites	Tissue regeneration, cartilage repair, cell culture, wound healing, skin regeneration, anti-aging treatments, bone repair, dental applications, bone regeneration, orthopedic implants	O’Brien et al. (2005); Gleeson, Plunkett & O’Brien (2010); Matsiko et al. (2012); Oryan et al. (2018); Calori et al. (2020)
		Elastin-based	-Elastin-like protein-hyaluronic acid (ELP-HA) hydrogel	Tissue repair, anti-aging treatments	Zhu et al. (2017)
		Alginate-based	-Alginate-chitosan composites -Alginate-calcium phosphate composites -Alginate-polymer nanocomposites - Alginate nanocomposites with carbon-based nanoparticles	Wound healing, drug delivery, tissue engineering, bone regeneration, dental applications, orthopedic implants	Wong (2004); Sun & Tan (2013); Goenka, Sant & Sant (2014); Venkatesan et al. (2015); Bibi, Rehman & Yaseen (2019)
		Chitosan-based	-Ceramic-chitosan composites	Bone repair, dental applications, orthopedic implants	Uskoković et al. (2021)
Synthetic scaffolding	Peptides based	Self-Assembling Peptide Scaffolds	-RADA16	Wound healing, neural tissue engineering, and angiogenesis	Wang et al. (2019)
			-MAX8	Tissue engineering, wound healing, biosensors, cancer research, 3D cell culture, angiogenesis, protein immobilization	Sperle, Pochan & Langhans (2023)
		Peptide Amphiphiles	-IKVAV Peptide Amphiphiles	Wound healing, neural regeneration, angiogenesis, cell signaling, 3D cell culture, stem cell differentiation, biomimetic materials, bioactive scaffold, glioma treatment, nerve repair, cell adhesion promotion, surgical implants, peripheral nerve regeneration, spinal cord injury repair, controlled cell proliferation	Wu & Lin (2022)
			-RDG Peptide Amphiphiles	Angiogenesis, cell adhesion, neural regeneration, bioactive scaffold, surgical implants, controlled cell proliferation	Xu et al. (2024a); Xu et al., (2024b)
		Hybrid Peptide Scaffolds	-Collagen-Peptide Conjugates	Bone regeneration, cartilage repair, ligament engineering, tendon repair, skin grafts, corneal repair, vascular grafts, dental implants, bioactive hydrogels, cosmetic applications, anti-aging treatments, muscle regeneration	Jayaraman et al. (2020)
			-Gelatin-Peptide Conjugates: PGAG (polydioxanone/poly (l-lactic acid)–gelatin–A5G81) occluders	Cardiac tissue repair, vascular grafts, neural regeneration, customized implants, hemostatic agents, corneal implants, cartilage repair, guided tissue regeneration, nerve conduits, hernia repair, bone defect fillers, reconstructive surgery, bioactive wound dressings	Kong et al. (2024)
		Electrospun Peptide Nanofibers	-Collagen-mimetic Peptide (CMP) Nanofibers -Silk-like Peptide Nanofibers, -Recombinant biomimetic polypep- tides (BMPPs) - Elastin-like Peptide Nanofibers - Peptide Amphiphile Nanofibers	3D cell culture, ligament regeneration, biosensors, controlled cell differentiation, nerve conduit repair, vocal fold regeneration, anti-fouling coatings, bioartificial pancreas, chemotherapy adjuvants, cardiac patch development, meniscus repair, bioactive dental fillers, tissue adhesives and sealants	Kong et al. (2020); Koga, Kingetsu & Higashi (2021); Rodriguez-Cabello et al. (2021)
		Hydrogel Peptide Scaffolds	-Fmoc-based Peptide Hydrogels -Fmoc-based RGD-functionalised peptide hydrogels –PEG_8_-(FY)3	Drug delivery, tissue engineering, biosensing, cell adhesion, tissue regeneration, wound healing, 3D cell culture, bioactive molecule incorporation	Diaferia et al. (2020)
			-Peptide-Polymer Conjugate Hydrogels	Controlled release, wound healing, regenerative medicine, 3D cell culture, drug encapsulation	Chimisso et al. (2020)
	Polymer based	Polyesters	-Polycaprolactone (PCL)	Bone scaffolding, wound healing, 3D printing, controlled release, medical devices, sutures	Koch et al. (2022)
			-Poly(lactic acid) (PLA)	Biodegradable implants, sutures, bone scaffolding, 3D printing, controlled release, wound healing, medical devices, biodegradable packaging, surgical staples	Alam et al. (2020); Zhou et al. (2021)
			-Poly(glycolic acid) (PGA)	Biodegradable implants, sutures, bone scaffolding, 3D printing, controlled release, wound healing, medical devices, tissue scaffolding, vascular grafts	Yeo et al. (2021)
		Polyalcohols	-Polyvinyl alcohol (PVA)	Wound dressings, contact lenses, drug delivery systems, medical implants, tissue adhesives, hydrogels, water-soluble films, controlled release, embolization agents, biodegradable packaging	Sun et al. (2022)
		Copolymers	(PLGA)	Biodegradable implants, sutures, bone scaffolding, 3D printing, controlled release, wound healing, medical devices, microencapsulation, stent coatings	Eslami et al. (2018); Wei et al. (2022)

For instance, tissue engineering produces artificial tissues and studies the mechanisms of behavior of different cell types in a variety of environments. For these purposes, biomaterials whose structure replicates the natural properties of the extracellular matrix are required (Xing et al., 2022). These properties include biocompatibility, a high moisture content that supports cell growth, a fibrillar structure that promotes cell adhesion, and several special characteristics, such as electrical conductivity, which is necessary to mimic cardiac tissue. Chalard et al. (2024) described a method for synthesizing a composite hydrogel meeting the above criteria. They used methylacryloyl gelatine, which is a denatured form of collagen, the main protein of the extracellular matrix, to prepare the gel. This gives the gel good biocompatibility and favors cell adhesion. By combining methylacryloyl gelatine with biocompatible supramolecular fibers made from a small self-organizing molecule derived from sugar (N-heptyl-D-galactonamide), the authors varied the properties of the composite material, such as Young’s modulus. Obtaining hydrogels of different elasticity allows modeling of various processes occurring in body tissues, such as fibrosis or vascularization (Chalard et al., 2024).

Ranamalla et al. (2024) created a strategy to synthesize a hydrogel based on hyaluronic acid modified by incorporating cysteine fragments into its structure. This favored the formation of a disulfide-linked cross-linked framework. By varying the degree of hyaluronic acid substitution, the rheological properties of the system can be varied, selecting the optimal properties for specific purposes, for example, for potential tissue engineering applications for joint treatment.

Silingardi et al. (2024) studied the effect of doping apatite biomaterials with potential applications for vertebrate bone replacement or repair with cobalt, manganese, and strontium ions on osteogenesis and angiogenesis. The presence of foreign ions in apatite materials was found to reduce their porosity and increase their compressive strength. Strontium, manganese, and calcium ions in biomaterials grown in contact with human mesenchymal stem cells stimulate their viability and activity, while cobalt ions have the opposite effect. All the mentioned materials have a positive effect on the expression of vascular endothelial growth factor and von Willebrand factor. The materials doped with strontium ions have the best effect.

Xu et al. (2024a), Xu et al. (2024b) developed a new type of implant by applying strontium-doped mesoporous active glass to the surface of well-known orthopedic implants made of polyetheretherketone. The surface modification allowed for the stimulation of osteogenic differentiation while suppressing the formation of osteoclasts due to the enhancement of cell adhesion processes. These results showed that biomaterials with a modified surface can promisingly promote bone healing, while unmodified materials, having a bioinert nature, prevent it.

Composite materials containing polylactide and hydroxyapatite have a tensile modulus of elasticity and hardness close to the parameters of trabecular bone, which determines their clinical application (Huolman & Ashammakhi, 2007). Polylactide can overcome the brittleness of hydroxyapatite, but its degradation products, which change the metabolism of immune cells, can stimulate an inflammatory reaction. Maduka et al. (2024), by controlling metabolic states by altering the glycolytic flux around the implanted composite biomaterial using inhibitors (*e.g.*, aminooxyacetic acid), have shown that this method can be promisingly used to create an environment favorable for bone regeneration.

## Scaffolds and Drug Carriers

Scaffolds and drug carriers composed of sophisticated biomaterials are pioneering the field of regenerative medicine and targeted drug delivery.

Scaffolds are injectables or implants that facilitate the delivery and controlled release of drugs, transcription factors, enzymes, antibodies, magnetic nanoparticles, cells, proteins, and genes into the body by providing protection in the body before releasing them (Dixon et al., 2016; Calori et al., 2020). Various forms of scaffolds for drug delivery are available in the market, including traditional 3D porous matrix, nanofibrous matrix, thermosensitive sol–gel transition hydrogel, and porous microsphere. Scaffolds can be fabricated using natural polymers like alginate, proteins such as collagen, gelatine, fibrin, and albumin, as well as synthetic polymers such as polyvinyl alcohol and polyglycolide. Additionally, bio ceramics, including hydroxyapatites and tricalcium phosphates, are commonly employed for this purpose (Garg et al., 2012). Drug carriers are specialized systems designed to transport therapeutic compounds for pharmaceutical, cosmetic, and nutraceutical applications to targeted areas within the body, enhancing the precision and efficacy of treatments (Trucillo, 2021).

These sophisticated structures serve as critical frameworks for tissue engineering, providing support for cell attachment, proliferation (Calori et al., 2020), and differentiation. Moreover, biomaterial-based drug carriers enhance therapeutic efficacy by delivering pharmaceuticals to specific sites within the body in a controlled manner. They can be classified according to types of administration, shape, and size, or mean dimensions. The interplay between material science and biological systems in designing these carriers holds immense potential for advancing medical treatments, promising improved patient outcomes through more efficient and localized therapeutic interventions.

From a biomaterials perspective, several types of drug carriers exist. Lipid-based nanocarriers include liposomes (Farooque, Wasi & Mughees, 2021), niosomes (Alyami et al., 2020), ethosomes (El Fawal et al., 2020), cubosomes (Zhai et al., 2020), and polymersomes (Travanut et al., 2022), polymer-based systems include micelles (Vinchurkar & Kuchekar, 2021), dendrimers (Rabiee et al., 2020), hydrogels (Dreiss, 2020), polymer-drug conjugates (Junyaprasert & Thummarati, 2023), and aquasomes (Shanmugam & Srinivasan, 2024), inorganic and solid nanostructures include nanoparticles (Xiao et al., 2022), nanotubes (Dubey et al., 2021), and quantum dots (Samimi, Ardestani & Dorkoosh, 2021), biological and cell-derived carriers include exosomes (Liang et al., 2021) and macrophages (Xia et al., 2020). These carriers can be engineered from various materials such as biodegradable polymers (*e.g.*, PLGA, PLA), lipids, proteins, and inorganic materials (*e.g.*, gold, silica). The choice of material depends on factors like biocompatibility, drug release profile, and the intended application. Biomaterials used for drug carriers are typically designed to be non-toxic, biodegradable, and capable of precise drug release to ensure optimal therapeutic outcomes (Kamaly et al., 2016).

## Morphology and Mechanical Strength

The interplay between the morphology and mechanical strength of biomaterials determines their biocompatibility, effectiveness, as well as other properties.

Several important aspects of the morphology of biomaterials include their surface topology, porosity, and particle size. The changes in these properties modulate their interactions with biological tissues, including cell adhesion, proliferation, and biocompatibility (Rahmany & Van Dyke, 2013). Cell adhesion and differentiation promote tissue integration, which can be accomplished by imitating the natural extracellular matrix through the use of nanostructured surfaces (Place, Evans & Stevens, 2009; Naqvi & McNamara, 2020). Additionally, nutrient and gas exchange, waste removal, as well as cell migration and scaffold attachment, can be controlled through porosity (Hernandez & Woodrow, 2022; Donati et al., 2024). Moreover, bioavailability and drug release rates can be directly influenced by the morphology of drug delivery systems, specifically the surface-to-volume ratio. More porous structures and smaller particles have a larger surface-to-volume ratio, which can increase the contact area with biological tissues and consequently improve drug delivery overall (Danhier et al., 2012; Kumeria, 2022).

Essential properties of biomaterials contributing to mechanical strength include elasticity, compressive strength, tensile strength, and fatigue resistance. These properties can be adjusted for the intended purpose of a biomaterial. For example, soft tissue biomaterials need to be flexible and thus require elasticity, while bone regeneration biomaterials require compressive strength (Meyers et al., 2008; Qu et al., 2019). The internal microstructure is another important aspect, since grain size and phase distribution can affect the mechanical behavior of a material (Wang et al., 2020b). Moreover, the mechanical stability of biomaterials is important for their longevity under different physiological conditions. This can influence the rate and consistency of drug release. Injectable hydrogels are an example of a biomaterial with desired properties for drug delivery: they exhibit flexibility, allowing them to withstand deformation, while simultaneously being able to gradually deliver drugs (Cabral & Moratti, 2011). A combination of several technologies, including nanoparticles and hydrogels, is also seeing success in the area of localized drug delivery (Gao et al., 2016; Kass & Nguyen, 2022). Hydrogel has also seen an application in the localized delivery of drugs for cancer treatment (Mikhail et al., 2023).

When designing a biomaterial, both morphology and mechanical strength have to be taken into consideration. Mechanical properties are closely linked to specific morphological traits since they rely on each other, and one can affect the other. For example, increased porosity, although providing better nutrient flow and cell proliferation, also reduces mechanical strength. This is why a balance between the two is necessary, and different properties are controlled during the design and fabrication (Rahmany & Van Dyke, 2013). An important milestone in biomaterial applications was the incorporation of nanotechnology, since it provides an additional level of control over the balance between morphology and mechanical properties. For instance, mimicking of the extracellular matrix is accomplished through the usage of nanofiber-based scaffolds that have enhanced mechanical strength as well as increased surface area for cell attachment (Place, Evans & Stevens, 2009). Moreover, biomaterials requiring both flexibility and high tensile strength can be designed using carbon nanotubes and graphene (Zheng et al., 2021).

## Critical Knowledge Gaps and Challenges in Personalized Biomaterial-Based Drug Delivery

Despite significant progress in biomaterial engineering and nanotechnology, several critical knowledge gaps continue to limit the realization of truly personalized drug delivery systems. One major gap lies in the translation of material-level optimization into patient-specific therapeutic outcomes. While numerous studies demonstrate controlled release, targeting efficiency, improved biocompatibility under standardized experimental conditions, relatively few address how inter-individual biological variability—such as immune status, disease heterogeneity, metabolic differences, or genetic background—modulates biomaterial performance *in vivo*. A second unresolved challenge concerns immune–biomaterial interactions, which remain insufficiently predictable across patient populations. Although surface modification strategies such as PEGylation, ligand functionalization, and bioactive coatings have been widely adopted to reduce immunogenicity, emerging evidence indicates that repeated administration, prior immune priming, and disease-specific immune dysregulation can substantially alter circulation time, biodistribution, and therapeutic efficacy. These effects are rarely incorporated into biomaterial design frameworks, highlighting a disconnect between the immunology-driven insights and material engineering. Manufacturing scalability represents another critical bottleneck. Many advanced biomaterial systems, particularly hybrid nanoparticles, stimulus-responsive carriers, and multifunctional scaffolds—exhibit high structural and compositional complexity that challenges reproducible, good manufacturing practices (GMP)-compliant production. Batch-to-batch variability, limited standardization of critical quality attributes, and insufficient long-term stability data collectively hinder regulatory approval and clinical adoption.

Importantly, personalization in current biomaterial research is often conceptual rather than operational. While the term “personalized drug delivery” is frequently invoked, most systems remain designed for population-averaged performance rather than adaptive or stratified deployment. There is a clear need for frameworks that integrate patient stratification, predictive biomarkers, and modular biomaterial design to enable practical customization without compromising safety or scalability.

Addressing these gaps requires a shift from platform-centric innovation toward biologically informed, patient-aware design strategies, supported by interdisciplinary collaboration between materials scientists, clinicians, immunologists, and regulatory experts. Without this transition, the clinical impact of advanced biomaterials will remain limited despite continued technological innovation.

## Future Directions

In the future, Biomaterial research needs to prioritize scalable/precision fabrication, long-term biocompatibility studies, and bio-responsive clinical practice.

Indeed, for example, precision personalized nanomedicine can be targeted to tumor microenvironments, deal with immune status, and be used in patient pharmacogenomics. Nanocarriers that are modified (size, surface ligands, degradability) to an individual diseased person can increase efficacy and reduce off-target toxicity (Dey, Hassan & Pandey, 2024). Recent reviews highlight strategies to engineer carriers to navigate the tumor microenvironment and immune barriers (Manzari et al., 2021). Another example is a stimulus-responsive biomaterial that could be organized to release the drug in a smart way. In fact, diverse drugs, nucleic acids, proteins, and enzymes can be released in response to pH, enzymes, redox state, temperature, ultrasound, or light, enabling on-demand, spatially precise dosing. Several recent comprehensive reviews cover design principles and *in vivo* examples (Mashele, 2025).

To support the translation of these advanced systems into clinical practice, greater emphasis must also be placed on Good Manufacturing Practices (GMP) and immunological stratification (Zia et al., 2024). GMP-compliant manufacturing ensures reproducibility, safety, and regulatory acceptance of increasingly complex and patient-tailored biomaterials, while immunological stratification enables the categorization of patients based on immune profiles, inflammatory status, and immune–biomaterial interactions. Integrating these considerations is essential for minimizing immune-related variability, optimizing therapeutic response, and advancing toward truly personalized drug delivery systems (Zia et al., 2024).

The future brings a chance for many patients to get more personalized treatment. Personalized medicine increasingly relies on biologics and nucleic-acid therapies (*e.g.*, neoantigen vaccines, *ex vivo* cell therapies). Nucleic acid–based personalized vaccines came with the advancements in molecular techniques used to identify neoantigens, antigen prediction methodologies, and the development of new vaccine platforms. To produce such vaccines, the sequencing of patient tumor samples is required, data analysis for antigen prediction is needed, and tailored vaccine manufacturing (Chi et al., 2024). Combining diagnostic with therapy, a term named theranostics, enables scientists to adjust doses of drugs based on the response. This shortens the time to effective therapy and reduces guesswork. The idea of “precision delivery” as a pillar complementing precision diagnostics and therapeutics is gaining increasing attention from the scientific world (Thakor, 2025). The future includes wearable patches and closed-loop delivery systems for chronic conditions such as diabetes, pain, hormones, integrating sensors with biomaterial reservoirs and micro actuators. This will enable automated and responsive dosing, adapted to a patient’s continuous physiological tracking (Puccetti et al., 2024).

Further, biomaterial research needs to prioritize more precise and scalable production through a structured roadmap emphasizing Quality-by-Design (QbD) principles, modular automation, and standardized processes (Ansari et al., 2024; Yang et al., 2025).

Initiatives like the BioFabUSA Technical Roadmap outline phased development (Garcia et al., 2018). First, this involves organizing and improving the controls over raw materials (for example, GMP-grade extracellular matrix components) and implementing real-time sensors to monitor critical process parameters (CPPs) like pH and cell viability (Mir et al., 2018). Secondly, the implementation of closed, automated systems such as the Tissue Foundry for scaffold manufacturing in unclassified environments is required (Labant, 2020). Furthermore, the incorporation of artificial intelligence (AI)-powered analysis and predictive modelling is necessary to speed up the transition from laboratory to clinical production volumes while minimizing batch variability (Mu’azzam et al., 2024).

In order to reduce the manual approach, the implementation requires cross-industry standards for bioinks and software compatibility to accelerate FDA pathways (Zhu, 2025).

Further, 3D bioprinting connects precise manufacturing with personalized medicine by facilitating the creation of patient-specific scaffolds that incorporate drug delivery systems, thereby directly enhancing the nanocarrier strategies previously mentioned (Liu et al., 2022; Zoghi, 2024). For example, using computed tomography (CT)-derived models, extrusion bioprinting produces bone scaffolds that are anatomically compatible, enabling customized porosity (200–500 µm) and mechanical stiffness (10–50 MPa), replicating native trabecular bone, and are infused with therapeutics such as antibiotics or growth factors for targeted release (Yu et al., 2024; Khan et al., 2025).

Connecting 3D bioprinting to the bioresponsive themes, scaffolds function as flexible platforms for theranostics in oncology and chronic diseases, incorporating sensors for real-time monitoring of pharmacokinetics (Tao et al., 2025). For example, in bone metastasis, printed polymer-ceramic composites (such as PCL-hydroxyapatite) are designed to release monoclonal antibodies or nucleic acids in response to specific enzymatic signals, thereby minimizing off-target effects while promoting osteogenesis (Yang et al., 2024; Demirdogen, Kaya & Ocakoglu, 2025). This unified extension integrates precision diagnostics, therapeutics, and delivery mechanisms, exemplified by neoantigen-loaded scaffolds utilized in immunotherapy. Therefore, to reduce the integration gaps, future efforts must prioritize hybrid nanosystems and regulatory harmonization, per emerging roadmaps projecting AI-optimized designs by 2030 (He & Zeng, 2025). Preclinical data show 3D-printed implants achieving 20-day sustained release with 98% matrix resilience akin to native bone, but clinical translation demands standardized CQAs for porosity and degradation (Sory et al., 2025; Gebeshuber et al., 2025). By involving these in closed-loop wearables or tumor grafts, personalized drug delivery systems transform into a new adaptive system, reducing uncertainty in dosing for ailments such as breast cancer or diabetes.

## Conclusions

Biomaterials have emerged as a central pillar in the evolution of personalized drug delivery, enabling a transition from conventional, systemically administered therapies toward highly targeted, adaptive, and patient-specific treatment strategies. As highlighted throughout this review, advances in material science, nanotechnology, and surface engineering have collectively expanded the functional capabilities of drug delivery systems, allowing for improved bioavailability, controlled release, reduced systemic toxicity, and enhanced therapeutic efficacy. The integration of bioactive coatings, injectable platforms, hybrid nanostructures, and stimulus-responsive systems underscores the growing sophistication of biomaterial-based approaches in addressing complex clinical needs.

Despite substantial progress, the clinical translation of advanced biomaterials remains constrained by challenges related to large-scale manufacturing, long-term biocompatibility, immunological interactions, regulatory approval, and cost-effectiveness. The complexity of next-generation biomaterials—while enabling multifunctionality and precision—also necessitates rigorous standardization, comprehensive safety evaluation, and harmonized regulatory frameworks. Addressing immune responses, degradation kinetics, and material reproducibility is essential to ensure consistent clinical outcomes and patient safety.

Looking forward, the convergence of biomaterials with precision medicine, artificial intelligence, and advanced manufacturing technologies such as 3D bioprinting is expected to redefine drug delivery paradigms. Future systems are likely to be dynamic and adaptive, capable of responding to physiological cues, disease progression, and individual patient profiles in real time. The integration of theranostic platforms, closed-loop delivery systems, and bioresponsive scaffolds holds particular promise for chronic disease management, oncology, and regenerative medicine. Ultimately, the successful implementation of biomaterial-based personalized drug delivery will depend on balancing innovation with ethical responsibility, regulatory compliance, and long-term clinical validation. By fostering interdisciplinary collaboration and aligning technological advances with patient-centered outcomes, biomaterials are poised to play an increasingly transformative role in precision healthcare, paving the way for safer, more effective, and sustainable therapeutic solutions.

## Additional Information and Declarations

### Data Availability

The following information was supplied regarding data availability: This is a literature review.
